# Histopathologic features of alcoholic cardiomyopathy compared with idiopathic dilated cardiomyopathy

**DOI:** 10.1097/MD.0000000000012259

**Published:** 2018-09-28

**Authors:** Xuebiao Li, Yu Nie, Hong Lian, Shengshou Hu

**Affiliations:** State Key Laboratory of Cardiovascular Disease, Fuwai Hospital, National Center for Cardiovascular Disease, Chinese Academy of Medical Sciences and Peking Union Medical College, Beijing, China.

**Keywords:** alcoholic cardiomyopathy, histopathology, idiopathic dilated cardiomyopathy

## Abstract

**Background:**

The histologic difference between alcoholic cardiomyopathy (ACM) and idiopathic dilated cardiomyopathy (IDCM) is unclear. The present study aimed to identify the quantitative pathologic features of ACM compared with IDCM.

**Methods:**

Specimens from 6 regions (anterior left ventricle [LV], lateral LV, inferior LV, interventricular septum [IVS], anterior right ventricle [RV], and inferior RV) were sampled from each explanted heart. Specimens from 4 healthy donor hearts were obtained as normal control. Tissues were sectioned and Masson trichrome stained. Histomorphometry was performed to evaluate the amount of myocyte, fibrosis, fatty tissue, and interstitium by Image-Pro Plus 6.0 (Media Cybernetics).

**Results:**

A total of 408 specimens were obtained from 34 ACMs and 34 IDCMs; 8 specimens were obtained from 4 healthy donor hearts. Compared to healthy donor hearts, we observed an increase in fibrosis which replaces myocytes in myocardium of end-stage cardiomyopathy. The overall myocyte ratio in myocardium was 69.5 ± 8.7% in ACM vs 71.9 ± 7.4% in IDCM (*P* < .05). The percentage of interstitium was 10.8 ± 4.8% in ACM vs 9.2 ± 6.2% in IDCM (*P* < .05). A significant difference of fibrosis, fatty tissue was not discovered. Moreover, the myocyte area was 65.37 ± 11.8% in ACM LV vs 70.03 ± 9.0% in IDCM LV (*P* < .001).

**Conclusion:**

We described histologic characteristics in ACM and IDCM. There might be a quantitative difference of myocyte, interstitium in myocardium between ACM and IDCM, especially in LV. No difference was found in the percentage of fibrosis between the 2 groups.

## 1 Introduction

Dilated cardiomyopathy (DCM) is a heart muscle disease characterized by progressive ventricular chamber enlargement and contractile dysfunction without increased left ventricle (LV) thickness.^[1,2]^ DCM is one of the most common reasons for heart failure.^[3]^ The 5-year mortality of DCM is approximately 50% after diagnosis.^[4]^ In most cases, the cause of DCM was unclear, defined as “idiopathic” dilated cardiomyopathy (IDCM). Up to 40% cases were speculated to be associated with chronic alcohol consumption, which was termed as alcoholic cardiomyopathy (ACM).^[4,5]^

In clinical practice, ACM is diagnosed by combining the dilated LV phenotype and a lengthy history of heavy alcohol abuse.^[5]^ However, any specific clinical characteristics, immunologic biomarkers, or other criteria are not yet identified for distinguishing ACM from IDCM. We collected transmural samples from 178 patients with DCM when they underwent heart transplantation during the past decade at Fuwai Hospital, China. Among them, approximately 18% were diagnosed as ACM, providing an opportunity for quantitative investigation of the pathologic characteristics of ACM.

The present study aimed to identify the histopathologic features of ACM compared to IDCM, which might be helpful to understand the development of ACM and differentiate it from IDCM.

## 2 Materials and methods

### 2.1 Patients

In a retrospective analysis, 280 patients with end-stage cardiomyopathies who underwent heart transplantation at Fuwai Hospital from June 2004 to June 2015 were screened. Patients were excluded if they demonstrated evidence of uncontrolled hypertension, history of myocardial infarction or coronary artery disease with >50% stenosis of one or more epicardial coronary arteries or other identified causes. The diagnosis of ACM was patient with DCM with no identified cause and a long history of heavy alcohol abuse (>80 g/d, at least 5 years). IDCM was defined as left ventricular dilatation with impaired systolic function in the absence of known cardiac or systemic causes.

Heart tissues from 4 healthy donors were chosen as normal controls. Their hearts were un-useful for transplantation due to blood-type incompatibility or other surgical reasons. These individuals died because of cerebrovascular or motor vehicle accident.

The present study was approved by the Committee on Human Investigation at Fuwai Hospital (Peking Union Medical College, China) conforming to the principles of the Helsinki Declaration.

### 2.2 Human myocardium tissue acquisition

The tissues used in our study were obtained from explanted failing human hearts at the time of heart transplantation.^[6]^ Full-thickness samples from 6 regions including anterior LV, lateral LV, inferior LV, IVS, anterior right ventricle (anterior RV), and inferior RV were acquired at the junction of base-mid ventricle of heart. In normal control group, full-thickness samples were obtained from 2 regions including left free ventricle wall and right free ventricle wall. Specimens were fixed in 10% neutral buffered formalin, embedded in paraffin, cut into 3 μm sections, and stained with Masson trichrome.

### 2.3 Image processing and data abstraction

Whole slide images were acquired by a microscopic digital scanner (Axio Scan Z1; Carl Zeiss, Oberkochen, Baden-Wuerttemberg, Germany) with 20× magnification. These scanned tiff image files are stored on the computer. We analyzed the pathologic images using Adobe Photoshop CS5 and Image-Pro Plus 6.0 (Media Cybernetics, Rockville, MD).^[7]^ The pixel intensity corresponding to myocyte, fibrosis, fatty tissue was acquired, respectively.^[8,9]^ The interstitium space could be calculated by substracting the readily identifiable myocyte, fibrosis, fatty tissue from the total tissue.^[10,11]^ The extent of myocyte, fibrosis, fatty tissue, and interstitium was presented as percentage of the whole examined section area.

### 2.4 Statistical analysis

Continuous variables were presented as mean ± standard deviation, and categorical variables were expressed as numbers (%). *T*-test was used when compared to normal healthy donor hearts. For comparison of the histologic difference between ACM and IDCM, the general linear model (GLM) was used. Discontinuous variables were compared using the Chi-squared test. The SPSS software (SPSS Inc, Chicago, IL) was used, and a 2-tailed *P* < .05 was considered statistically significant.

## 3 Results

### 3.1 Characteristics of included patients

Among the 280 cases, 102 patients were excluded from our analysis because they had identified as being caused by known disease: ischemia (n = 41), valvular disease (n = 5), hypertensive cardiomyopathy (n = 25), congenital heart disease (n = 1), inflammatory disease (n = 19), left ventricular noncompaction disease (n = 3), arrhythmogenic right ventricular cardiomyopathy (n = 2), and end-stage hypertrophic cardiomyopathy (n = 6), 178 patients were with no identified causes. Among the 178 cases, 34 cases were confirmed as ACM based on their medical history (chronic alcohol exposure, >80 g/d, >5 years). Another 34 patients with IDCM were selected for histomorphometrical comparison, matched by age and sex. A statistically significant difference was not observed in the clinical, laboratory examination, echocardiographic, and medical treatment data between the 2 group except for body mass index and total bilirubin level, which was moderately higher in patients with ACM compared to patients with IDCM (*P* < .05) (Table 1). In patients with ACM, the daily amount of ethanol intake ranged from 112 to 360 g/d (mean, 174.5 g/d), and the duration of alcohol abuse ranged from 6 to 30 years (mean, 15 years). The beverage choices were beer coupled with spirits (4, 11.8%), wine coupled with spirits (1, 2.9%), and spirits only (29, 85.3%).

**Table 1:**
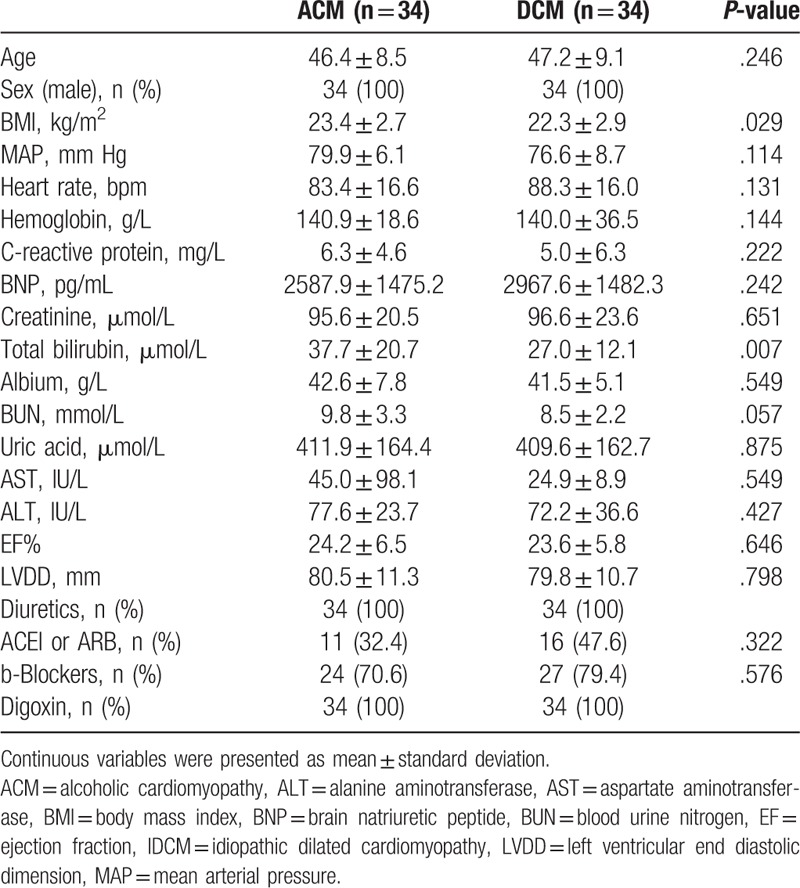
Basic clinical characteristics of included patients.

### 3.2 Histopathologic changes in end-stage cardiomyopathy

**Figure 1:**
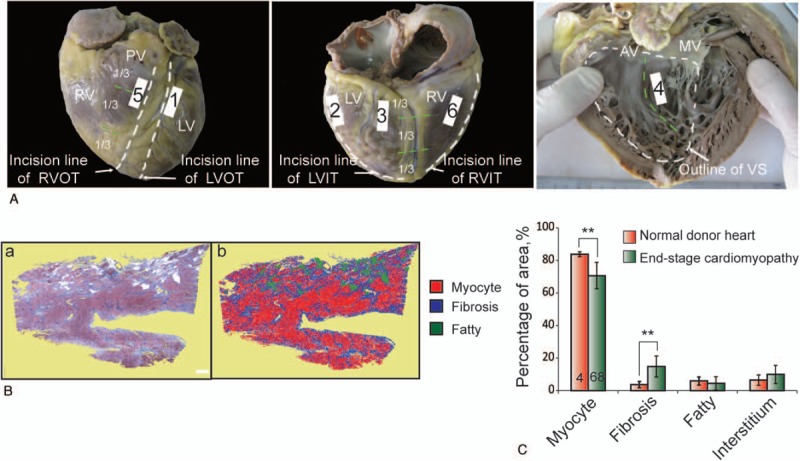
(A) Graphical representation of the sampling sites in resected disease heart. (B) Example of image processing and data abstraction for histologic evaluation of myocyte, fibrosis, fatty tissue, and interstitium. (a) Original Masson trichrome staining. (b) Illustration of the standard thresholding procedure at 20× magnification: areas occupied by red pixels (myocyte), blue pixels (fibrosis), and green pixels (identified fatty tissue) in photomicrograph assessed by image-processing software. The interstitium space could be calculated by substracting the readily identifiable myocyte, fibrosis, fatty tissue from the total tissue. Scale bars = 1 mm. (C) Compared to normal healthy donor hearts, we observed an increase in fibrosis which replaces myocytes in myocardium of end-stage cardiomyopathy. AV, aortic valve; IVS, inter-ventricle septum; LV, left ventricle; LVIT, left ventricular inflow tract; LVOT, left ventricle outflow tract; MV, mitral valve; RV, right ventricle; RVIT, right ventricle inflow tract; RVOT, right ventricle outflow tract.

A total of 416 Masson trichrome-stained slides were histomorphometrically evaluated (Fig. 1 A, B). The quantitative measurements of myocyte, fibrosis, fatty tissue, and interstitium in myocardium of disease hearts are showed in Table 2.

**Table 2:**
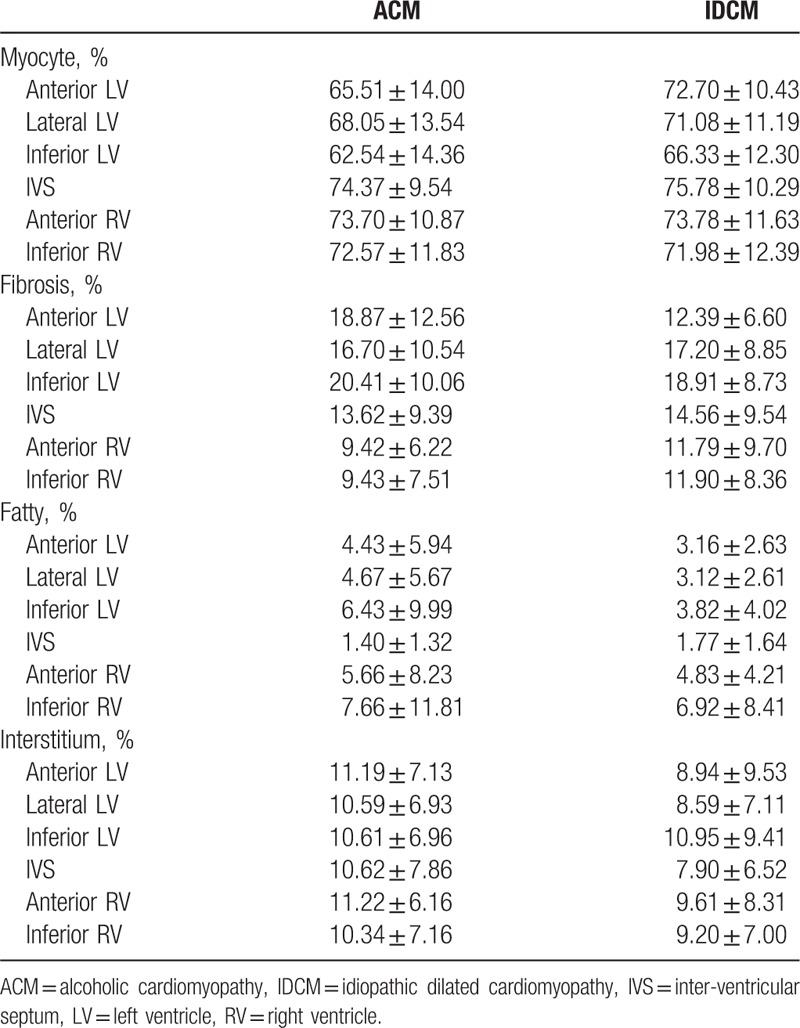
Histopathologic data of myocyte, fibrosis, fatty, and interstitium in the explanted hearts corresponding to 6 sampling sites from patients with ACM and IDCM.

In myocardium of normal donor hearts, the cumulative amount of myocyte was 83.92 ± 1.50%, of fibrosis was 3.67 ± 1.93%, of fatty tissue was 5.94 ± 2.49%, of interstitium was 6.47 ± 3.17%. The amount of myocyte area was higher in LV than that in RV; the amount of fibrosis, fatty tissue was lower in LV than that in RV; no significant difference was observed in interstitium between LV and RV (Table 3).

**Table 3:**
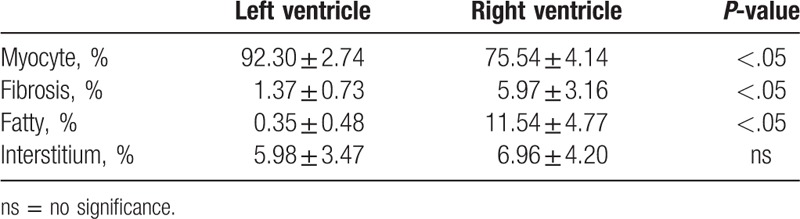
Quantitative measurement related to left ventricle or right ventricle in normal controls.

Compared to normal healthy donor hearts, we observed an increase in fibrosis which replaces myocytes in myocardium of end-stage cardiomyopathy (14.83% ± 9.71% vs 3.67 ± 1.93%, *P* < .01; 70.70% ± 12.43% vs 83.92 ± 1.50%, *P* < .01, respectively) (Fig. 1C).

### 3.3 Quantitative comparison of the histomorphometric parameters between ACM and IDCM

**Figure 2:**
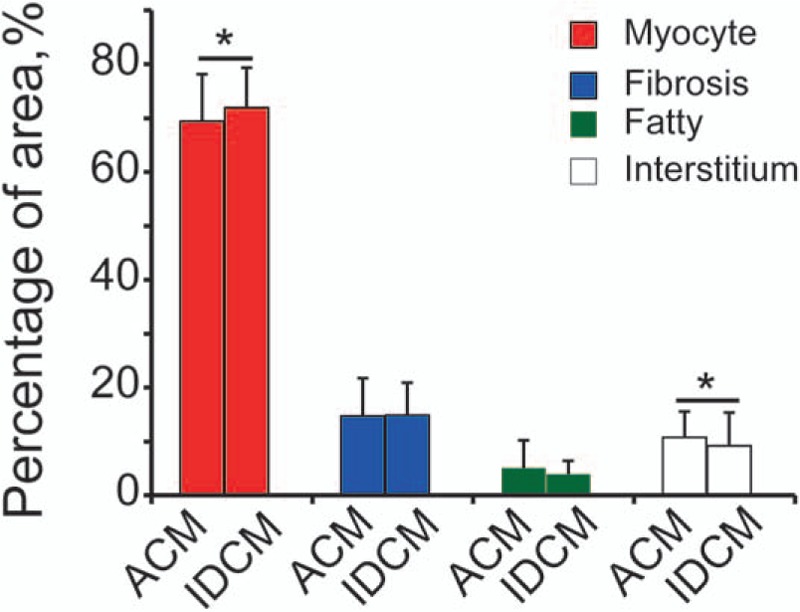
Cumulative analysis of percentage area of myocyte, fibrosis, fatty tissue, and inerstitium in alcoholic cardiomyopathy (ACM) compared with idiopathic dilated cardiomyopathy (IDCM). Myocyte area between ACM and IDCM was significantly different. The general linear model was used. Dependent variables: myocyte, fibrosis, fatty tissue or interstitium; fixed factors: groups (ACM, IDCM); positions (anterior left ventricular [LV], lateral LV, inferior LV, interventricular septum, anterior right ventricular [RV], inferior RV). No significant difference was observed in amount of fibrosis, fatty tissue. ∗*P* < .05.

To elucidate the histomorphometric differences between ACM and IDCM, we performed comparison based on the cumulative measurements (composition of 6 sampling regions). We found a significant difference in the percentage area of myocyte and interstitium between ACM and IDCM (69.5 ± 8.7% vs 71.9 ± 7.4%, *P* < .05; 10.8 ± 4.8% vs 9.2 ± 6.2%, *P* < .05, respectively). The amount of fibrosis and fatty tissue was detected no quantitative difference (Fig. 2).

**Figure 3:**
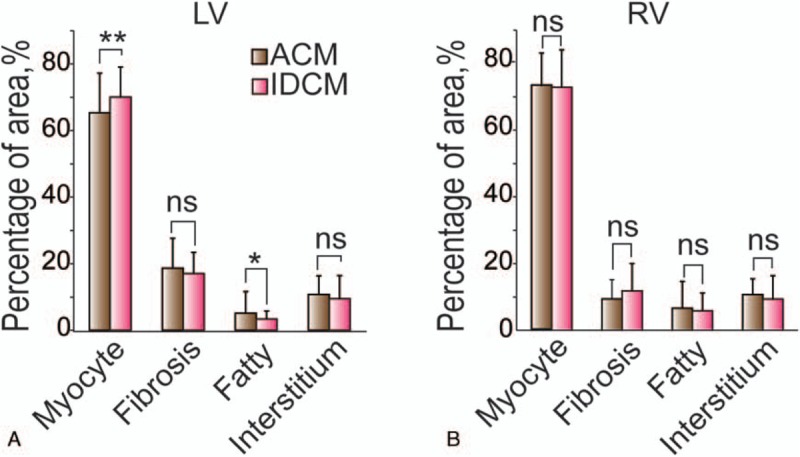
Ventricle-based analysis of histologic difference between alcoholic cardiomyopathy (ACM) and idiopathic dilated cardiomyopathy (IDCM). (A) A significant reduction of myocyte was observed in ACM left ventricular (LV) compared to that in IDCM. (B) Based on measures related to right ventricular (RV), we found no histomorphologic differences between ACM and IDCM. The general linear model was used. Dependent variable: myocyte, fibrosis, fatty tissue or interstitium; fixed factor: group (ACM, IDCM); position (anterior LV, lateral LV, inferior LV, interventricular septum, anterior RV, inferior RV). ∗*P* < .05, ∗∗*P* < .01. ns = no significance.

Furthermore, we detected a significant reduction of myocyte in ACM LV compared to that in IDCM (65.37 ± 11.83% vs 70.03 ± 9.02%, *P* < .01) (Fig. 3A). Based on measures related to RV, we found no histomorphologic differences between ACM and IDCM (Figure 3 B). These results showed histologic alternations in LV might have greater value to that in RV for distinguishing ACM from IDCM.

## 4 Discussion

To the best of our knowledge, this is the first and the largest cohort of ACM patients investigated for the histologic characteristics compared to IDCM using full-thickness cardiac specimens. The analysis of specimens in 68 patients revealed a reduction of myocyte in ACM compared with IDCM.

Since the introduction of the term “alcoholic cardiomyopathy” in 1902, diagnosis for ACM is mainly based on a history of long-term heavy alcohol intake without abnormal loading conditions (hypertension, valve disease) or coronary artery disease and certain drugs (doxorubicin, cocaine), or other certain disease.^[5,12]^ Several studies had revealed a complex myriad of histologic changes in ACM including different degrees of cardiomyocyte atrophy, interstitial and perivascular fibrosis, hyperplasia of small mitochondria, widening and winding of Z-discs, intracristal inclusions (circled) of mitochondria, dehiscence of intercalated disks, and large vacuoles in cardiomyocytes.^[13–16]^ In previous study, they reported milder changes in myocytic hypertrophy, fibrosis compared to IDCM using samples obtained by endomyocardial biopsy (EMB).^[17]^ Although EMB is considered to be safe in patients with cardiomyopathies, the size of the biopsy is about 2 to 5 mm^3^, leading great variability when comparing 2 biopsies from the same ventricle, and may not reveal the ventricle full-thickness changes.^[18,19]^ In present study, we took advantage of transmural myocardial specimens avoiding the endocardial–epicardial distribution bias. The size of transmural sample in our study is approximately 0.6 to 0.8 cm^3^, making it more reliable when compared with EMB specimens in evaluation of histopathologic changes. We found there was a quantitative difference of myocyte, interstitium in myocardium between ACM and IDCM.

In a previous study, extent of interstitial fibrosis was found higher in patients with DCM with end-stage HF than in tissue samples without heart disease (autopsy cases) (4.97 ± 0.83% vs1.12 ± 0.18%, *P* < .05).^[20]^ Our result was consistent with this finding. Moreover, they also reported that fibrosis differed among ACM and IDCM (10.77 ± 2.09% vs 4.97 ± 0.83%, *P* < .05).^[20]^ This result was inconsistent with our data. In DCM, the percentage of fatty tissue was 0.07 ± 0.31%, the percentage of interstitium was 14.55 ± 3.93%. There was no statistical difference of interstitium between DCM and controls (EMB from hearts 7 days after transplantation).^[10]^ In our results, the percentage of fatty tissue was high. Our analysis was based on transmural specimens, the endocardial–epicardial distribution bias may contribute to this difference. In previous study, they reported the extent, distribution of ventricular pathologically characteristics varied between LV and RV. The histologic changes were maximally involved in LV, while right ventricle was usually spared. Also, in our study, we found histologic alternations in LV were more remarkable and valuable to that of RV in ACM and IDCM.

### 4.1 Limitations

Notably, there were several limitations in our study. First, population size in our study was modest, results of our study should be further validated in a larger cohort of patients. Second, in our study, patients with ACM were male, because of heavy drinking is common among men in China. Further study should be conducted to detect the effect of sex and race on the histologic characteristics. Finally, these samples were collected from patients with end-stage heart failure, so results could not reflect alcohol induced toxic effect on heart in early phase. But available transmural samples could only be collected from patients with heart failure when they receiving heart transplantation.

In conclusion, we firstly described the histopathologic aspects in ACM based on transmural tissues. Our results provided insight into the histologic changes in ACM and IDCM.

## Author contributions

**Data curation:** Yu Nie.

**Formal analysis:** Yu Nie.

**Funding acquisition:** Yu Nie, Shengshou Hu.

**Investigation:** Xuebiao Li.

**Methodology:** Yu Nie, Shengshou Hu.

**Project administration:** Yu Nie.

**Resources:** Shengshou Hu.

**Supervision:** Shengshou Hu.

**Validation:** Hong Lian.

**Writing – original draft:** Xuebiao Li.

**Writing – review & editing:** Hong Lian, Shengshou Hu.
